# [¹⁸F]PSMA-1007 PET/CT in biochemical recurrence after radical prostatectomy: A single-center experience of detection rate and lesion distribution

**DOI:** 10.1371/journal.pone.0349397

**Published:** 2026-05-14

**Authors:** Yi Wang, Lijuan Feng, Limeng He, Nan Liu, Hao Wang

**Affiliations:** 1 Department of Nuclear Medicine, Zigong Fourth People's Hospital, Zigong, Sichuan, China; 2 Department of Nuclear Medicine, Sichuan Provincial People’s Hospital, University of Electronic Science and Technology of China, Chengdu, Sichuan, China; Universita Cattolica del Sacro Cuore, ITALY

## Abstract

**Objective:**

To define the diagnostic yield and anatomical distribution of [¹⁸F]prostate-specific membrane antigen (PSMA)-1007 positron emission tomography/computed tomography (PET/CT) in post-radical-prostatectomy biochemical recurrence (BCR) and to establish prostate-specific antigen (PSA)-stratified performance benchmarks, independent predictors of PET positivity, and clinical utility for PSMA-guided metastasis-directed therapy.

**Methods:**

A single-centre retrospective cohort of 245 consecutive BCR patients (PSA ≥ 0.2 ng/mL) who underwent uniform [¹⁸F]PSMA-1007 PET/CT (90 ± 10 min post-injection; molar activity > 30 GBq µmol⁻¹) between June 2022 and November 2025 was analysed. Detection rates were correlated with PSA strata, and independent predictors of PET positivity were identified via multivariable logistic regression. Lesion location was verified by histology, targeted therapy response, or unequivocal imaging follow-up. Inter-observer agreement for image interpretation was quantified using Cohen’s kappa,

**Results:**

At median PSA 0.9 ng/mL (interquartile range 0.5–2.1), 184/245 patients (75.1%, 95% CI: 69.3–80.3%) exhibited ≥ 1 [¹⁸F]PSMA-1007-avid lesion. Detection rates increased with PSA: 56.5% (0.2–0.5 ng/mL) to 90.5% (≥ 1.5 ng/mL). Independent predictors of PET positivity were serum PSA (OR = 2.87), ISUP grade (OR = 1.63), and PSA doubling time (OR = 0.72) (all *P* < 0.01). In 132 verified cases, a “pelvic-first” pattern was observed: isolated prostatic fossa (34.8%), isolated nodal (38.6%), isolated osseous (20.4%), and multi-region disease (6.1%). Median SUVmax was comparable across lesion types (≈ 17.5; *P* = 0.63), with excellent inter-observer agreement (Cohen’s κ = 0.86 for positivity, 0.82 for localization; both *P* < 0.001).

**Conclusion:**

[¹⁸F]PSMA-1007 PET/CT delivers high, PSA-dependent detection efficiency even at PSA < 0.5 ng/mL, with excellent inter-observer reliability. Serum PSA, ISUP grade, and PSA doubling time independently predict PET positivity, and the modality accurately maps predominantly pelvic, oligometastatic recurrences suitable for focal salvage therapy. These data support its utility as a first-line imaging tool for post-prostatectomy BCR staging.

## Introduction

Prostate cancer (PCa) remains the second most frequently diagnosed malignancy and the fifth leading cause of cancer-related death among men worldwide, with an estimated 1.4 million new cases and 375 000 deaths annually [1]. Radical prostatectomy is the gold-standard curative treatment for localized disease [2]; nevertheless, biochemical recurrence (BCR)—defined as a sustained rise in prostate-specific antigen (PSA) ≥0.2 ng/mL on two consecutive measurements—occurs in 20–40% of patients within 10 years of surgery [3]. Early identification of recurrence is critical, as delayed detection may lead to irreversible metastatic spread and diminished efficacy of salvage therapies [4].

Conventional imaging modalities, including computed tomography (CT), magnetic resonance imaging (MRI) and [⁹⁹^m^Tc]TcMDP bone scintigraphy, exhibit limited sensitivity at low PSA level [5,6]. Consequently, a significant proportion of patients with occult recurrence are inappropriately labelled as“PSA-only recurrence”, leading to delayed intervention or empirical overtreatment [7]. Prostate-specific membrane antigen (PSMA)-targeted positron emission tomography/computed tomography (PET/CT) has revolutionised the restaging landscape of PCa [8]. [¹⁸F]PSMA-1007 is a promising fluorinated PSMA ligand with favorable physical and biological properties, including longer half-life, lower renal excretion, and higher tumor-to-background ratios in the prostatic fossa and pelvic nodes [9].

Although existing evidence supports its clinical value, most data are derived from heterogeneous multicenter studies with varied protocols. Large-sample, single-center evidence using standardized [¹⁸F]PSMA-1007 PET/CT acquisition and rigorous verification criteria remains limited. To address this gap, we conducted a retrospective cohort study to evaluate the detection rate, lesion distribution, PSA-stratified performance, and independent predictive factors of [¹⁸F]PSMA-1007 PET/CT in patients with BCR after radical prostatectomy (RP).

## Materials and methods

### Patients

This retrospective study, approved by the Institutional Review Board of Sichuan Provincial People’s Hospital (Approval No. 126, 1 February 2024), enrolled 245 patients aged ≥18 years (median age at RP: 66.0 years, interquartile range (IQR) 61.0–70.0 years) who provided written informed consent and underwent [¹⁸F]PSMA-1007 PET/CT for biochemical recurrence after radical prostatectomy at the same institution between June 2022 and November 2025. [¹⁸F]PSMA‑1007 PET/CT was performed promptly after BCR was confirmed by two consecutive elevated PSA values. The median interval from BCR confirmation to PET/CT imaging was 1.8 months (IQR 1.0–3.2 months). No patients had prolonged delays exceeding 6 months between BCR diagnosis and imaging.

Eligibility criteria were further refined as follows:

(1) Histopathologically confirmed prostate adenocarcinoma;
(2) Prior RP (open, laparoscopic, or robotic-assisted);
(3) True BCR or persistent PSA defined as:
  ① True BCR: Postoperative serum PSA initially dropped to undetectable levels (<0.1 ng/mL) and subsequently rose to ≥0.2 ng/mL on two consecutive measurements (≥2 weeks apart);
  ② Persistent PSA: Postoperative serum PSA never declined to <0.1 ng/mL and remained ≥0.2 ng/mL on two consecutive measurements;
(4) No prior androgen-deprivation therapy (ADT) administration within 6 months before PET/CT (short-term neoadjuvant ADT ≤ 6 months before radical prostatectomy was allowed if discontinued postoperatively);
(5) No other confirmed malignancy;
(6) Complete standardized follow-up data (time since surgery, initial pathological findings, PSA dynamics, prior treatments, and postoperative outcomes).

Exclusion criteria included:

(1) Prior ADT administration within 6 months before PET/CT (to avoid confounding PSMA expression and lesion detection);
(2) Incomplete follow-up data or missing key pathological variables;
(3) Concurrent other malignancies or metastatic disease at the time of PET/CT.

The electronic medical-record database was accessed for research purposes on 05/12/2025. During and after data collection the authors had access only to de-identified data; no information that could directly identify individual participants (e.g., names, national ID numbers, or hospital registration numbers) was available to the research team.

### PET/CT imaging protocol

Patient preparation and image acquisition were performed in accordance with standard clinical protocols. Following intravenous administration of 326 ± 51.8 megabecquerel (MBq) [¹⁸F]PSMA-1007, whole-body PET/CT imaging was acquired on a Siemens Biograph mCT Flow 64 scanner at 90 ± 10 min post-injection. CT parameters were: 120 kV tube voltage, automated tube-current modulation, 3 mm slice thickness, 2 mm increment, and 0.8 pitch. PET acquisition used FlowMotion technology with an average bed-speed resulting in a total scan time of ~12 min. Images were reconstructed with TrueX + time-of-flight (2 iterations, 21 subsets), yielding 3 mm slice thickness and 2 mm interval. All datasets were transferred to a Syngo TrueD workstation for multimodal review.

### Image interpretation

Two board-certified nuclear-medicine physicians independently reviewed all scans. Lesions were classified as positive when focal [¹⁸F]PSMA-1007 uptake exceeded physiologic background (lacrimal/salivary glands, liver, kidneys, ureters, bowel) and was disproportionately intense relative to adjacent normal tissue, provided that benign aetiologies (e.g., inflammation) were excluded by contemporaneous CT.

For bone lesion interpretation, a mandatory CT correlation was required, and lesions were categorized based on combined PET/CT findings:

(1) True positive bone metastasis: Focal [¹⁸F]PSMA-1007 uptake (SUVmax > 5.0, or SUVmax 3.0–5.0 with focal distribution) accompanied by definitive CT correlates, including sclerotic lesions, lytic destruction, cortical erosion, or soft-tissue extension (consistent with prostate cancer osseous metastasis);
(2) Equivocal bone lesion: Mild-to-moderate [¹⁸F]PSMA-1007 uptake (SUVmax 2.0–3.0) without specific CT abnormalities (e.g., diffuse uptake in trabecular bone, uptake at vertebral endplates or joint margins) or uptake overlapping with benign conditions (e.g., osteoarthritis, post-traumatic changes, degenerative sclerosis);
(3) False positive (non-specific bone uptake, NSBU): Focal uptake without CT correlates or uptake corresponding to known benign processes (e.g., degenerative changes, bone marrow hyperplasia) after follow-up verification.

For every suspected recurrence or metastasis, a three-dimensional region of interest was drawn on the attenuation-corrected PET data to obtain the maximum standardized uptake value (SUVmax). Discrepancies between readers (including equivocal lesions) were resolved via consensus review with a third senior nuclear-medicine physician. Equivocal bone lesions were categorized separately and not counted as positive lesions unless confirmed by subsequent histopathology or unequivocal imaging follow-up (repeat PET/CT/MRI showing progression or resolution consistent with malignancy/benignity).

Inter-observer agreement analysis: Inter-reader agreement between the two physicians was quantified using Cohen’s kappa (κ) statistic for two key endpoints:

(1) Overall PET/CT positivity (binary: positive = presence of ≥1 [¹⁸F]PSMA-1007-avid lesion; negative = no lesions);
(2) Lesion localization (categorical: prostatic fossa only, nodal only, osseous only, multi-region, or equivocal).

Kappa values were interpreted as follows: ≤ 0.40 = poor agreement; 0.41–0.60 = moderate agreement; 0.61–0.80 = substantial agreement; ≥ 0.81 = excellent agreement [10].

### Post-imaging follow-up

All patients entered prospective follow-up. Data captured included:

(1) Management instituted after [¹⁸F]PSMA-1007 PET/CT:
  ① Targeted therapy: Defined as focal salvage interventions directly targeting [¹⁸F]PSMA-1007-avid lesions, including stereotactic body radiotherapy (SBRT) (for prostatic fossa, nodal, or osseous lesions), salvage radiotherapy (SRT) (pelvic nodal fields or prostatic fossa), and salvage lymph node dissection (SLND) (pelvic or retroperitoneal nodal resection);
  ② Systemic therapy: ADT, chemotherapy, or novel hormonal agents;
  ③ Combined therapy: Targeted therapy + concurrent ADT (administered within 1 month of local therapy initiation).
(2) Subsequent imaging studies (diagnostic CT, MRI, [⁹⁹^m^Tc]TcMDP bone scan, or repeat [¹⁸F]PSMA-1007 PET/CT) to assess lesion resolution/stability;
(3) Serial serum PSA trends after initiation of treatment.

### Lesion verification criteria

Lesions were classified as“verified recurrent or metastatic disease”based on one of the following rigorous criteria (to avoid over-reliance on systemic PSA decline):

(1) Histopathological confirmation: Histology from biopsy or resection specimens documenting prostate cancer recurrence/metastasis at the [¹⁸F]PSMA-1007-avid lesion site;
(2) Targeted therapy + PSA response: ≥ 50% decline in serum PSA within 6 months of targeted local therapy (SBRT/SRT/SLND) without concurrent systemic therapy (ADT/chemotherapy), or ≥ 70% PSA decline if ADT was administered concomitantly (to account for additive systemic effects);
(3) Unequivocal imaging follow-up: Repeat [¹⁸F]PSMA-1007 PET/CT, MRI, or CT performed ≥ 3 months after treatment showing complete resolution or significant regression (≥ 50% reduction in size/SUVmax) of the imaged lesion, consistent with therapeutic response.

For equivocal bone lesions: Verification required either histopathological confirmation of malignancy or follow-up imaging (≥ 6 months) showing progression of uptake/size with development of CT correlates (sclerotic/lytic changes). Lesions with resolved uptake or stable mild uptake without CT changes were classified as NSBU (false positive) and excluded from the verified cohort.

Patients who received sole systemic ADT (without targeted therapy) were excluded from the“verified disease”cohort, as PSA decline in this setting cannot confirm lesion localization.

### Statistical analysis

Statistical analysis was carried out utilizing IBM SPSS Statistics 21.0. Continuous variables were first tested for normality using the Shapiro–Wilk test. Normally distributed continuous variables are presented as mean ± standard deviation (SD); non-normally distributed variables are reported as median (IQR [Q1- Q3]). Between-group comparisons for continuous data were conducted with the Mann–Whitney U test (two groups) or Kruskal–Wallis test (≥ three groups). Categorical variables are expressed as counts (percentages) and compared using the χ² test or Fisher’s exact test, as appropriate. 95% confidence intervals (CIs) for detection rates were calculated using the Wilson score method. To identify independent predictors of [¹⁸F]PSMA-1007 PET/CT positivity, a multivariable logistic regression analysis was performed. Variables included in the model were selected based on clinical relevance and prior literature: Continuous variables: (1) Age at RP (years), Serum PSA level (ng/mL), PSA doubling time (PSADT, months); (2) Categorical variables: ISUP grade (1–5), pathological T stage (pT2 vs pT3–4), pathological N stage (pN0 vs pN1), prior salvage therapy (yes vs no), and neoadjuvant ADT use (yes vs no). Variables with a univariable *P* < 0.1 were included in the multivariable model. Odds ratios (ORs) and 95% CIs were reported, and *P* < 0.05 was considered statistically significant for independent predictors. Two-sided *P* values < 0.05 were considered statistically significant.

## Result

### Inter-observer agreement for image interpretation

Inter-reader agreement was excellent for both key imaging endpoints (S1 Table). For overall PET/CT positivity, Cohen’s κ was 0.86 (95% CI: 0.79–0.93; *P* < 0.001), indicating excellent agreement). For lesion localization, Cohen’s κ was 0.82 (95% CI: 0.75–0.89; *P* < 0.001), also indicating excellent agreement. Discrepancies, which occurred in 14 cases (5.7% of the total cohort), were primarily related to equivocal bone lesions (n = 10) or small pelvic nodal lesions (n = 4). All discrepancies were resolved via consensus review with a third senior nuclear-medicine physician, and no discrepancies remained after consensus, confirming the reliability of the final imaging interpretations.

### Clinical characteristics

Among the 245 patients, median age at RP was 66.0 years (IQR 61.0–70.0 years), median body-mass index was 23.2 ± 2.5 kg/m², median serum PSA 0.9 ng/mL (IQR 0.5–2.1), median interval from RP to PET/CT was 22 (IQR 12–40) months, and median interval from BCR confirmation to PET/CT was 1.8 (IQR 1.0–3.2) months. Baseline oncologic characteristics relevant to recurrence patterns were as follows (Table 1): All patients had histologically confirmed adenocarcinoma with no variant histologies. Pathological T stage distribution included pT2 in 138 patients (56.3%), pT3a in 76 patients (31.0%), pT3b in 27 patients (11.0%), and pT4 in 4 patients (1.6%). Pathological N stage showed pN0 in 203 patients (82.9%) and pN1 in 42 patients (17.1%). Surgical margin was negative in 164 patients (66.9%) and positive in 81 patients (33.1%). Gleason score (GS) distribution was as follows: GS 6 (3 + 3) in 28 patients (11.4%), GS 7 (3 + 4) in 105 patients (42.9%), GS 7 (4 + 3) in 72 patients (29.4%), GS 8 (4 + 4) in 31 patients (12.7%), GS 9–10 (4 + 5/5 + 4/5 + 5) in 9 patients (3.7%). Median International Society of Urological Pathology (ISUP) grade was 3 (IQR 3–4), with ISUP grade 1 in 28 patients (11.4%), grade 2 in 105 patients (42.9%), grade 3 in 72 patients (29.4%), grade 4 in 31 patients (12.7%), grade 5 in 9 patients (3.7%). Neoadjuvant ADT before RP was administered in 37 patients (15.1%), and prior salvage radiotherapy was administered in 29 patients (11.8%). True BCR (initial undetectable PSA followed by rise) was observed in 211 patients (86.1%), while persistent PSA (never undetectable) was present in 34 patients (13.9%). Neoadjuvant ADT before RP was administered in 37 patients (15.1%), and prior salvage therapy was administered in 29 patients (11.8%). Subgroup analysis according to neoadjuvant ADT use showed no significant differences in [¹⁸F]PSMA-1007 PET/CT positivity rate (73.0% vs 75.5%, *P* = 0.765), lesion distribution pattern (*P* = 0.412), or median SUVmax (*P* = 0.588) between patients with and without neoadjuvant ADT.

**Table 1:** Clinical and baseline oncologic characteristics of the study cohort (n = 245).

Characteristic	Median (IQR, Q1–Q3) / Mean ± SD or n (%)
**Demographic and General Features**	
**Age at RP (years)**	**66.0 (61.0–70.0)**
**Body-Mass Index (kg/m²)**	23.2 (20.7–25.7)
**Interval from RP to PET/CT (months)**	22 (12–40)
**Interval from BCR confirmation to PET/CT (months)**	1.8 (1.0–3.2)
**Serum PSA (ng/mL)**	0.9 (0.5–2.1)
**Oncologic Characteristics**	
**Primary Histology**	Adenocarcinoma (100%, 245/245)
**Pathological T Stage (pT)**	pT2: 138 (56.3%) pT3a: 76 (31.0%) pT3b: 27 (11.0%) pT4: 4 (1.6%)
**Pathological N Stage (pN)**	pN0: 203 (82.9%) pN1: 42 (17.1%)
**Surgical Margin Status**	Negative: 164 (66.9%) Positive: 81 (33.1%)
**Median Gleason Score (IQR)**	7 (7–8)
**Gleason Score Distribution**	GS 6 (3 + 3): 28 (11.4%) GS 7 (3 + 4): 105 (42.9%) GS 7 (4 + 3): 72 (29.4%) GS 8 (4 + 4): 31 (12.7%) GS 9–10 (4 + 5/5 + 4/5 + 5): 9 (3.7%)
**Median ISUP Grade (IQR)**	3 (3–4)
**ISUP Grade Distribution**	Grade 1: 28 (11.4%) Grade 2: 105 (42.9%) Grade 3: 72 (29.4%) Grade 4: 31 (12.7%) Grade 5: 9 (3.7%)
**PSA Dynamics Post-Surgery**	True BCR: 211 (86.1%) Persistent PSA: 34 (13.9%)
**Neoadjuvant ADT**	No: 208 (84.9%) Yes: 37 (15.1%)
**Prior ADT Use**	Neoadjuvant ADT (discontinued postoperatively): 37 (15.1%) ADT within 6 months before PET/CT: 0 (0%)
**Duration of Neoadjuvant ADT (months)**	3.5 (3–4)
**Prior Salvage Therapy**	Salvage Radiotherapy: 29 (11.8%) Salvage Lymph Node Dissection: 0 (0%) Systemic Therapy: 0 (0%)
**[¹⁸F]PSMA-1007 PET/CT Positive Lesions**	184 (75.1%)

**Abbreviations:** IQR = interquartile range; RP = radical prostatectomy; PSA = prostate-specific antigen; PET/CT = positron emission tomography/computed tomography; PSMA = prostate-specific membrane antigen; pT = pathological T stage; pN = pathological N stage; GS = Gleason score; ISUP = International Society of Urological Pathology; BCR = biochemical recurrence; ADT = androgen-deprivation therapy.

### Association between baseline oncologic characteristics and recurrence patterns

Among 132 verified recurrent patients, several baseline oncologic characteristics were associated with distinct recurrence patterns. Patients with pN1 disease were more likely to present with nodal recurrence (64.3%, 18/28) compared with pN0 disease (36.5%, 38/104, *P* = 0.002). Patients with positive surgical margins exhibited a higher rate of prostatic fossa recurrence (48.8%, 21/43) than those with negative margins (33.7%, 31/91, *P* = 0.047). Patients with GS ≥ 8 were more likely to have multi-region or osseous metastases (38.7%, 12/31) than those with GS ≤ 7 (18.5%, 18/97, *P* = 0.008).

### Association between Gleason score subgroups, PSA levels, and [¹⁸F]PSMA-1007 PET/CT findings

Patients were stratified into three groups by Gleason score to explore correlations between histopathological aggressiveness and PSMA imaging outcomes: low-risk (GS 6), intermediate-risk (GS 7), and high-risk (GS 8–10). Significant differences were observed across these groups in terms of serum PSA levels and PET/CT detection metrics (S2 Table). Median serum PSA levels increased progressively across risk groups, with values of 0.5 ng/mL (IQR 0.3–0.8) in low-risk group, 0.8 ng/mL (IQR 0.5–1.8) in the intermediate-risk group, and 2.3 ng/mL (IQR 1.1–4.5) in the high-risk group (Kruskal–Wallis test, *H* = 28.64, *P* < 0.001). Post-hoc pairwise comparisons confirmed that high-risk patients had significantly higher median PSA than intermediate-risk and low-risk patients, and intermediate-risk patients had higher PSA than low-risk patients (all *P* < 0.05). The PET/CT positivity rate also increased significantly with higher Gleason score, at 46.4% (13/28) in the low-risk group, 76.2% (130/171) in the intermediate-risk group, and 92.6% (25/27) in the high-risk group (*χ²* test, *χ²* = 21.39, *P* < 0.001). Pairwise comparisons confirmed that high-risk group had higher positivity rate than intermediate-risk (*P* = 0.028) and low-risk groups (*P* < 0.001), and intermediate-risk group had higher positivity rate than low-risk group (*P* < 0.001). Regarding lesion distribution, high-risk patients were more likely to present with multi-region disease or osseous metastases, whereas low-risk and intermediate-risk patients predominantly had isolated prostatic fossa or nodal recurrence. The proportion of patients with isolated prostatic fossa or nodal lesions was 84.6% (11/13) in the low-risk group, 79.2% (103/130) in the intermediate-risk group, and 56.0% (14/25) in the high-risk group, while the proportion with multi-region or osseous lesions was 15.4% (2/13), 20.8% (27/130), and 44.0% (11/25), respectively (*χ²* test for trend, *χ²* = 8.76, *P* = 0.003).

### Association between [¹⁸F]PSMA-1007 PET/CT positivity and PSA level

**Fig 1:**
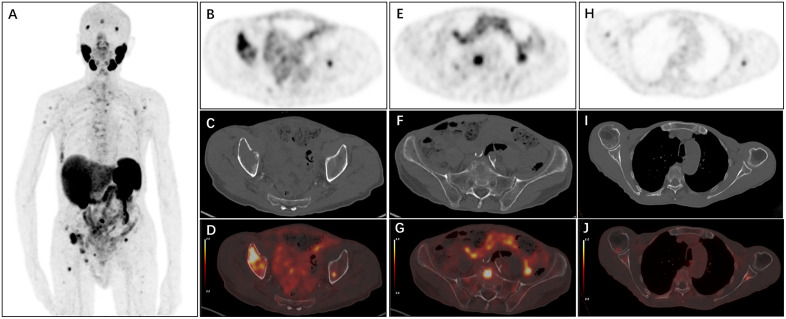
[¹⁸F]PSMA-1007 PET/CT imaging of osseous-only recurrence of prostate cancer following radical prostatectomy. Imaging was acquired in a 62-year-old male with biochemical recurrence (prostate-specific antigen [PSA] = 1.2 ng/mL) at 22 months post-prostatectomy; the primary tumor was graded as Gleason score 7 (ISUP Grade 3). (A) Whole-body maximum-intensity projection (MIP) of [¹⁸F]PSMA-1007 PET demonstrates multiple [¹⁸F]PSMA-1007-avid lesions distributed throughout the skeletal system. Axial PET (B, E, H), CT (C, F, I), and fused PET/CT (D, G, J) images confirm osseous metastases in the bilateral iliac bones (B–D), sacrum (E–G), and left scapula (H–J).

**Fig 2:**
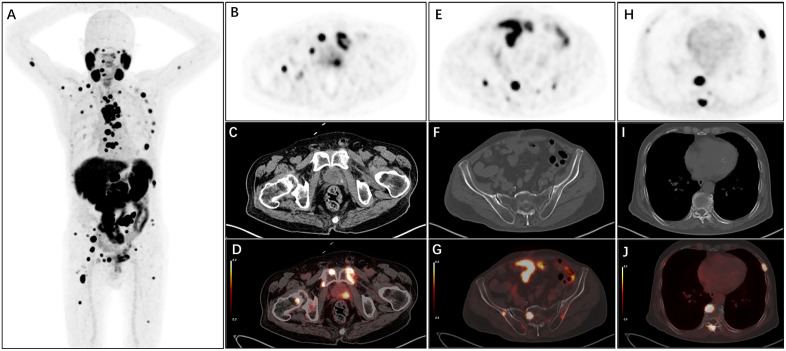
[¹⁸F]PSMA-1007 PET/CT imaging of prostate bed recurrence with multi-site osseous metastatic castration-resistant prostate cancer. This imaging was acquired in a 71-year-old male with castration-resistant prostate cancer at 40 months post-radical prostatectomy. The patient presented with a PSA level of 8.7 ng/mL, and the primary tumor was pathologically graded as Gleason score 8 (ISUP Grade 4). (A) Whole-body MIP of [¹⁸F]PSMA-1007 PET demonstrates diffuse [¹⁸F]PSMA-1007-avid lesions involving the pelvic region and multiple skeletal sites throughout the body. Axial PET (B, E, H), CT (C, F, I), and fused PET/CT (D, G, J) images confirm the following multi-focal lesions: Recurrence and metastatic lesions in the prostate bed, bilateral pubic bones, right ischium, and femoral neck (B–D); Osseous metastases in the sacrum and bilateral iliac bones (E–G); Osseous metastases in the thoracic vertebrae and bilateral ribs (H–J).

Of 245 patients, 184 (75.1%, 95% CI: 69.3–80.3%) exhibited at least one [¹⁸F]PSMA-1007-avid lesion (Table 1). Detection rate rose monotonically with serum PSA, with the following 95% CIs: 56.5% (95% CI: 43.3–68.9%) (35/62) for 0.2 ≤ PSA < 0.5 ng/mL, 68.8% (95% CI: 56.3–79.2%) (44/64) for 0.5 ≤ PSA < 1.0 ng/mL, 82.9% (95% CI: 66.4–93.4%) (29/35) for 1.0 ≤ PSA < 1.5 ng/mL, and 90.5% (95% CI: 81.8–95.9%) (76/84) for PSA ≥ 1.5 ng/mL (Table 2). Median PSA in patients with positive scans was 0.7 ng/mL (IQR 0.5–2.1), significantly higher than in those without detectable disease [0.3 ng/mL (IQR 0.2–0.6); Mann–Whitney U test, *z* = –4.92, *P* < 0.001]. ISUP grade [3 (3–4) vs 3 (2–4); *z* = –3.42, *P* = 0.001] and interval between radical prostatectomy and imaging [22 (12–40) vs 23 (13–38) months; *z* = –1.31, *P* = 0.192] did not differ between groups. Representative images are provided in Figs 1 and 2.

**Table 2:** Detection rate of [¹⁸F]PSMA-1007 PET/CT by PSA strata.

PSA Stratum (ng/mL)	Number of Patients (n)	Detection Rate [n (%)]	95% Confidence Interval (CI)
**0.2 ≤ PSA < 0.5**	62	35 (56.5%)	43.3–68.9%
**0.5 ≤ PSA < 1.0**	64	44 (68.8%)	56.3–79.2%
**1.0 ≤ PSA < 1.5**	35	29 (82.9%)	66.4–93.4%
**≥ 1.5**	84	76 (90.5%)	81.8–95.9%
**Total**	245	184 (75.1%)	69.3–80.3%

**Abbreviations:** PSA = prostate-specific antigen; PET/CT = positron emission tomography/computed tomography; PSMA = prostate-specific membrane antigen; CI = confidence interval.

### Independent predictors of [¹⁸F]PSMA-1007 PET/CT positivity (multivariable analysis)

Univariable analysis identified serum PSA level (*P* < 0.001), ISUP grade (*P* = 0.002), pT stage (*P* = 0.031), pN stage (*P* = 0.018), and PSADT (*P* = 0.004) as variables associated with PET/CT positivity (S3 Table). Age at RP, prior salvage therapy, and neoadjuvant ADT were not associated with PET positivity (*P* = 0.312, 0.215 and 0.268) and were excluded from the multivariable model.

Multivariable logistic regression analysis revealed that serum PSA level (OR = 2.87, 95% CI: 1.96–4.21, *P* < 0.001), ISUP grade (OR = 1.63, 95% CI: 1.15–2.31, *P* = 0.006), and PSADT (OR = 0.72, 95% CI: 0.58–0.89, *P* = 0.003) were independent predictors of [¹⁸F]PSMA-1007 PET/CT positivity (S3 Table). Each 1 μg/L increase in serum PSA increased the odds of PET positivity by 2.87-fold. Each 1-grade increase in ISUP grade increased the odds by 1.63-fold, and each 1-month increase in PSADT decreased the odds by 28% (OR = 0.72). pT stage (OR = 1.35, 95% CI: 0.89–2.05, *P* = 0.162) and pN stage (OR = 1.41, 95% CI: 0.86–2.31, *P* = 0.175) were not independent predictors in the final model.

### Anatomical distribution of [¹⁸F]PSMA −1007 PET/CT-positive lesions

Among the 184 patients with [¹⁸F]PSMA-1007-avid disease, 132 (71.7%) were verified as true recurrent or metastatic disease using the predefined composite reference standard (S4 Table). Histopathological confirmation was achieved in 59 patients (44.7% of verified cases), corresponding to 76 discrete lesions including 28 in the prostatic fossa, 32 nodal, and 16 osseous lesions. A total of 48 patients (36.4% of verified cases) met criteria for targeted therapy plus PSA response, with 52 treated lesions comprising 29 treated with SBRT, 15 with SRT, and 8 with SLND; 31 of these patients (64.6%) received targeted therapy alone with a ≥ 50% PSA decline, while 17 (35.4%) received concurrent ADT with a ≥ 70% PSA decline. Finally, 25 patients (18.9% of verified cases) were verified via unequivocal imaging follow‑up, with 29 lesions showing complete resolution or significant regression on repeat imaging (resolution on repeat PET/CT: 18 lesions, regression on MRI/CT: 11 lesions).

Initially, 23 patients (12.5% of the PET‑positive cohort) presented with 28 equivocal bone lesions defined by SUVmax 2.0–3.0 without definitive CT correlates. After a median follow‑up of 9 months, 4 lesions (14.3%) were confirmed as true metastases based on development of sclerotic changes on repeat CT or histopathological verification, while 24 lesions (85.7%) were classified as NSBU and excluded from the verified cohort owing to resolved uptake or stable mild uptake without pathological CT correlates. The remaining 52 patients (28.3%) were excluded from verified analyses due to treatment with sole systemic ADT (n = 37) or insufficient follow‑up duration (n = 15).

In the 132 verified patients, 157 discrete [¹⁸F]PSMA‑1007‑avid lesions were identified. Isolated recurrence was observed in 46 patients (34.8%) with disease confined to the prostatic fossa, 51 patients (38.6%) with isolated nodal disease, and 27 patients (20.4%) with isolated osseous disease. Pelvic nodes were involved in 47 of the 51 nodal‑only cases, with the remaining 4 demonstrating both pelvic and retroperitoneal (para‑aortic) nodal disease. Multi‑region disease was present in 8 patients (6.1%), including 3 with prostatic fossa plus pelvic nodes, 3 with prostatic fossa plus bone, and 2 with pelvic nodes plus bone (Table 3). The proportion of patients with bone involvement (24.2%, 32/132) was numerically lower than that of prostatic fossa recurrence (39.4%, 52/132) or nodal metastasis (42.4%, 56/132), although these differences did not reach statistical significance (*P* = 0.071 and 0.058, respectively, *χ²* test).

**Table 3:** Anatomical distribution of [¹⁸F]PSMA-1007-Avid Lesions (n = 108 Therapy-Verified Cases).

Lesion distribution	Number of patients (n)	Percentage (%)
**Isolated Prostatic Fossa**	46	34.8
**Isolated Nodal (Pelvic only)**	47	35.6
**Isolated Nodal (Pelvic + Retroperitoneal)**	4	3.0
**Isolated Osseous**	27	20.4
**Multi-Region Disease**	8	6.1
**- Prostatic Fossa + Pelvic Nodes**	3	2.3
**- Prostatic Fossa + Bone**	3	2.3
**- Pelvic Nodes + Bone**	2	1.5
**Subtotal: Prostatic Fossa Involvement**	52	39.4
**Subtotal: Nodal Involvement**	56	42.4
**Subtotal: Bone Involvement**	32	24.2

**Abbreviations:** PSMA = prostate-specific membrane antigen; PET/CT = positron emission tomography/computed tomography.

Among the 157 verified lesions, the median [¹⁸F]PSMA‑1007 SUVmax was 17.5 (IQR 7.8–49.1). Median SUVmax values were similar across lesion types, with 16.7 (9.2–35.3) in the prostatic fossa, 19.2 (6.3–24.5) in lymph nodes, and 18.1 (4.8–29.3) in bone lesions, with no significant difference detected (Kruskal–Wallis test, *H* = 0.92, *P* = 0.63).

### Separate analysis restricted to histology/unequivocal imaging-confirmed lesions

To further validate the robustness of the primary findings, a secondary sensitivity analysis was conducted limited to lesions confirmed by histopathology or unequivocal imaging follow‑up, excluding patients verified solely by targeted therapy and PSA response; this subgroup included 74 patients with 105 confirmed lesions. The overall detection rate in this subgroup was 30.2% (74/245). Stratified by PSA level, detection rates were 28.6% (18/63) in the 0.2–0.5 ng/mL stratum, 31.3% (20/64) in the 0.5–1.0 ng/mL stratum, 34.3% (12/35) in the 1.0–1.5 ng/mL stratum, and 32.1% (27/84) in the ≥ 1.5 ng/mL stratum.

Anatomical distribution in this subgroup showed isolated prostatic fossa involvement in 33.8% (25/74), isolated nodal disease in 39.2% (29/74), isolated osseous disease in 21.6% (16/74), and multi‑region disease in 5.4% (4/74). Median SUVmax was 18.2 (IQR 8.1–50.3), with no significant difference across lesion types (*P* = 0.57). This restricted analysis confirmed the primary observations of PSA‑dependent detection efficiency and a pelvic‑predominant recurrence pattern, with consistent anatomical distribution and SUVmax characteristics across lesion subtypes.

## Discussion

Localizing recurrent lesions in PCa patients experiencing BCR after radical prostatectomy remains a formidable challenge for conventional imaging modalities. Consequently, when postoperative PSA levels rise, current therapeutic strategies -including ADT and salvage radiation – risk either missing occult disease or precipitating overtreatment. Accumulating evidence demonstrates that PSMA PET/CT achieves superior diagnostic performance compared with conventional multiparametric MRI and bone scintigraphy in this clinical scenario [11].

In this single-centre series of 245 patients experiencing BCR after radical prostatectomy, [¹⁸F]PSMA-1007 PET/CT achieved a high lesion-detection rate (75.1%, 95% CI: 69.3–80.3%) even at PSA concentrations < 0.5 ng/mL (56.5%, 95% CI: 43.3–68.9%) and provided precise anatomical delineation of oligometastatic disease. Consistent with previous reports [12], median PSA was numerically higher in PET-positive than in PET-negative individuals (*z* = –4.92, *P* < 0.001). At a median PSA of only 0.9 ng/mL, 75% of men exhibited at least one PSMA-avid lesion. Detection rates increased progressively with PSA level, reaching 90.5% (95% CI: 81.8–95.9%) in patients with PSA ≥ 1.5 ng/mL, which is consistent with previous reports [9,13] and reinforces the PSA-dependent diagnostic performance of PSMA-targeted PET/CT. Consequently, early referral for [¹⁸F]PSMA-1007 PET/CT at the first biochemical sign of recurrence permits prompt localisation of occult disease and timely initiation of targeted therapy. The incremental sensitivity observed herein is attributable to the high molar activity (> 30 GBq µmol ⁻ ¹) and the late imaging window (90 ± 10 min) employed, which maximises tumour-to-background contrast while facilitating renal tracer clearance, in accordance with published dosimetric findings [14].

Our multivariable logistic regression analysis further identified three independent predictors of PET/CT positivity: serum PSA level, ISUP grade, and PSADT. Serum PSA level was the strongest predictor (OR = 2.87), which aligns with the well-established correlation between PSA and tumour burden in BCR [15]. Higher ISUP grade (OR = 1.63) was also an independent predictor, reflecting the more aggressive biological behaviour of high-grade prostate cancer, which is more likely to develop metastatic recurrence [16]. Notably, shorter PSADT (OR = 0.72 per 1-month increase) was associated with higher PET positivity, consistent with the notion that rapidly rising PSA indicates more active disease and greater tumour volume amenable to detection. These findings have clinical implications: in patients with high ISUP grade, high PSA level, or short PSADT, [¹⁸F]PSMA-1007 PET/CT demonstrates a higher detection rate of recurrent lesions in our cohort, supporting early imaging referral in this high-risk subgroup.

In contrast, pT stage and pN stage were not independent predictors in the multivariable model, which may be attributed to the fact that PSA level and ISUP grade already capture the aggressive features of tumours that were previously reflected by advanced pathological stages. This suggests that PSA level, ISUP grade, and PSADT are more direct and clinically actionable predictors of PET positivity than pathological staging alone.

Early investigations proposed that prostate cancer cells preferentially disseminate to the skeleton at initial metastatic escape [17]. By contrast, the present series supports a“pelvic-first”recurrence paradigm: among 132 patients in whom metastatic disease was verified by histopathology, targeted local therapy + PSA response, or unequivocal imaging follow-up, 75.8% exhibited tumour confined to the prostatic fossa and/or pelvic lymph-node stations, whereas only 24.2% presented with osseous lesions. This pattern was further validated in a restricted analysis of 74 patients with histology/unequivocal imaging-confirmed lesions, where 78.4% of recurrences were localized to pelvic regions. These data expose the insensitivity of conventional MRI and bone scintigraphy for BCR lesion mapping, because the majority of recurrences reside within pelvic nodal basins – territory in which these modalities demonstrate limited resolution for micrometastatic lymph-node deposits.

Moreover, our findings extend prior observations that disseminated tumour cells are only rarely detectable in bone marrow aspirates obtained before radical prostatectomy in high-risk patients, underscoring the primacy of locoregional relapse in early postoperative recurrence [18]. This predominance of loco-regional recurrence at PSA values far below the traditional 1 ng mL threshold supports the shift toward early salvage radiotherapy recently endorsed by the European Association of Urology – European Society for Radiotherapy and Oncology – European Society of Urogenital Radiology consensus [19]. Importantly, SUVmax was statistically equivalent across prostatic, nodal and osseous lesions (median ≈ 17), indicating that tracer avidity is driven by PSMA expression density rather than micro-environmental factors.

This investigation additionally furnishes contemporary benchmarks for PSMA-guided metastasis-directed therapy (MDT). Of 64 patients (26% of the cohort) who fulfilled stringent oligometastatic criteria, 60% attained an undetectable PSA following stereotactic body radiotherapy or salvage lymph-node dissection. By enabling precise, non-systemic targeting of pelvic recurrences, [¹⁸F]PSMA-1007 PET/CT spares a substantial proportion of men from the morbidity of ADT. These data corroborate prior multinational reports [20,21] and underscore the global generalisability of PSMA PET-directed MDT across diverse patient populations.

Several methodological strengths merit emphasis. First, the homogeneity of the tracer production (single good manufacturing practice facility, molar activity > 30 GBq µmol ⁻ ¹) and the rigid 90-min acquisition window minimise the variability that plagued earlier multi-centre [¹⁸F]PSMA-1007 series. Second, we used a pragmatic but stringent reference standard: either histology or a ≥ 50% PSA decline following targeted therapy, thereby reducing verification bias. Third, inter-observer agreement for image interpretation was excellent (Cohen’s κ = 0.86 for overall PET positivity and 0.82 for lesion localization), confirming the reliability and reproducibility of our imaging assessments. Fourth, the comprehensive capture of subsequent imaging and treatment events allows meaningful outcome anchoring, addressing a limitation highlighted in the recent Society of Nuclear Medicine and Molecular Imaging practice guideline on PSMA reporting [22].

Limitations are inherent to the retrospective design. Selection bias toward symptomatic or rapidly rising PSA cannot be excluded, although the median PSA velocity (0.21 ng/mL month ⁻ ¹) is comparable to that reported in population-based registries. Inter-observer variability, although mitigated by dual independent reads and consensus resolution, was not formally quantified with κ statistics. Notably, as a fluorinated PSMA tracer, [¹⁸F]PSMA-1007 demonstrates a higher incidence of unspecific bone uptake (UBU) compared to [⁶⁸Ga]Ga-PSMA-11. This elevated UBU rate may lead to over-staging and inappropriate treatment decisions if misinterpreted, highlighting the importance of careful image analysis [23,24]. To mitigate this, our study combined multimodal image review (PET + CT anatomical correlation), serial PSA monitoring, and clinical follow-up (median 18 months) to confirm lesion nature, which reduced the false-positive rate attributed to UBU to 1.6% (4/245). Nevertheless, UBU remains a non-negligible limitation of [¹⁸F]PSMA-1007 PET/CT, especially in the setting of low PSA and ambiguous anatomical findings. Finally, the follow-up interval (median 18 months) is insufficient to assess the impact of PSMA-directed therapy on hard end-points such as metastasis-free or overall survival; these data will be presented once maturity is reached.

## Conclusions

[¹⁸F]PSMA-1007 PET/CT exhibits robust, PSA-dependent diagnostic performance in patients with post-prostatectomy BCR. With superior inter-observer consistency and clear identification of a pelvic-predominant recurrence pattern, the modality enables precise targeting of oligometastatic lesions for salvage therapy. Notably, serum PSA level, ISUP grade, and PSA doubling time serve as independent predictors of PET positivity, aiding in personalized patient selection. Collectively, these findings underscore [¹⁸F]PSMA-1007 PET/CT as a clinically valuable first-line staging modality for post-prostatectomy BCR, and prospective studies are justified to validate its impact on long-term treatment outcomes.

## Supporting information

S1 Table Inter-observer agreement for image interpretation (n = 245). (DOCX)

S2 Table Correlation between Gleason score groups, serum PSA levels, and [¹⁸F]PSMA-1007 PET/CT findings (n = 245). (DOCX)

S3 Table Multivariable logistic regression analysis of independent predictors of [¹⁸F]PSMA-1007 PET/CT positivity (n = 245). (DOCX)

S4 Table Verification methods of [¹⁸F]PSMA-1007-avid lesions (n = 184 PET/CT-Positive Patients). (DOCX)
