# Supplementary material for: [¹⁸F]PSMA-1007 PET/CT in biochemical recurrence after radical prostatectomy: A single-center experience of detection rate and lesion distribution
Source: PLoS One. 2026 May 14;21(5):e0349397. doi: 10.1371/journal.pone.0349397 (PMC13175320; doi:10.1371/journal.pone.0349397)
Supplement: S1 Table — (DOCX) [file pone.0349397.s001.docx]

**Table S1.** **Inter-Observer Agreement for Image Interpretation (n=245).**

| **Imaging Endpoint** | **Cohen’s Kappa (κ)** | **95% Confidence Interval (CI)** | ***P* Value** | **Agreement Category** |
| --- | --- | --- | --- | --- |
| **Overall PET/CT Positivity (Positive/Negative)** | 0.86 | 0.79–0.93 | < 0.001 | Excellent |
| **Lesion Localization (Prostatic Fossa/Nodal/Osseous/Multi-Region/Equivocal)** | 0.82 | 0.75–0.89 | < 0.001 | Excellent |

**Note:** Kappa interpretation: ≥0.81 = excellent agreement; 0.61–0.80 = substantial agreement; 0.41–0.60 = moderate agreement; ≤0.40 = poor agreement. Discrepancies (n=14) were resolved via consensus review with a third senior nuclear-medicine physician.
