# Supplementary material for: [¹⁸F]PSMA-1007 PET/CT in biochemical recurrence after radical prostatectomy: A single-center experience of detection rate and lesion distribution
Source: PLoS One. 2026 May 14;21(5):e0349397. doi: 10.1371/journal.pone.0349397 (PMC13175320; doi:10.1371/journal.pone.0349397)
Supplement: S2 Table — (DOCX) [file pone.0349397.s002.docx]

**Table S2.** **Correlation between Gleason Score Groups, Serum PSA Levels, and [¹⁸F]PSMA-1007 PET/CT Findings (n=245).**

| **Parameter** | **Low-Risk (GS 6)**  **n=28 (%)** | **Intermediate-Risk (GS 7)**  **n=171 (%)** | **High-Risk (GS 8–10)**  **n=27 (%)** | **Statistical Test** | ***P* Value** |
| --- | --- | --- | --- | --- | --- |
| **Median PSA (IQR), μg/L** | 0.5 (0.3–0.8) | 0.8 (0.5–1.8) | 2.3 (1.1–4.5) | Kruskal–Wallis H | < 0.001 |
| **PET/CT Positive Lesions, n (%)** | 13 (46.4) | 130 (76.2) | 25 (92.6) | *χ²* Test | < 0.001 |
| **Lesion Distribution, n (%)** |  |  |  |  |  |
| **- Isolated Prostatic Fossa/Nodal** | 11 (84.6) | 103 (79.2) | 14 (56.0) | *χ²* Test for Trend | 0.003 |
| **- Multi-Region/Osseous** | 2 (15.4) | 27 (20.8) | 11 (44.0) |  |  |

**Abbreviations:** GS = Gleason score; PSA = prostate-specific antigen; IQR = interquartile range; PET/CT = positron emission tomography/computed tomography.

**Note:** Pairwise comparisons: For PSA, high-risk > intermediate-risk > low-risk (all *P* < 0.05); for PET positivity, high-risk > intermediate-risk > low-risk (all *P* < 0.05); for lesion distribution, high-risk had higher proportion of multi-region/osseous lesions than intermediate-risk and low-risk (all *P* < 0.05).
