# Supplementary material for: [¹⁸F]PSMA-1007 PET/CT in biochemical recurrence after radical prostatectomy: A single-center experience of detection rate and lesion distribution
Source: PLoS One. 2026 May 14;21(5):e0349397. doi: 10.1371/journal.pone.0349397 (PMC13175320; doi:10.1371/journal.pone.0349397)
Supplement: S3 Table — (DOCX) [file pone.0349397.s003.docx]

**Table S3.** **Multivariable Logistic Regression Analysis of Independent Predictors of [¹⁸F]PSMA-1007 PET/CT Positivity (n=245)**

| **Variable** | **Univariable Analysis**  **OR (95% CI)** | **Univariable P Value** | **Multivariable Analysis**  **OR (95% CI)** | **Multivariable P Value** |
| --- | --- | --- | --- | --- |
| **Age at RP (per 1-year increase)** | 0.99 (0.96–1.02) | 0.312 | — | — |
| **Serum PSA Level (per 1 ng/mL increase)** | 3.12 (2.18–4.46) | < 0.001 | 2.87 (1.96–4.21) | < 0.001 |
| **ISUP Grade (per 1-grade increase)** | 1.75 (1.24–2.47) | 0.002 | 1.63 (1.15–2.31) | 0.006 |
| **pT Stage (pT3–4 vs pT2)** | 1.89 (1.06–3.36) | 0.031 | 1.35 (0.89–2.05) | 0.162 |
| **pN Stage (pN1 vs pN0)** | 2.07 (1.14–3.75) | 0.018 | 1.41 (0.86–2.31) | 0.175 |
| **PSA Doubling Time (PSADT, per 1-month increase)** | 0.68 (0.54–0.85) | 0.004 | 0.72 (0.58–0.89) | 0.003 |
| **Neoadjuvant ADT (yes vs no)** | 0.86 (0.42–1.76) | 0.268 | — | — |
| **Prior Salvage Therapy (yes vs no)** | 1.32 (0.81–2.15) | 0.215 | — | — |

**Abbreviations:** OR = odds ratio; CI = confidence interval; RP = radical prostatectomy; PSA = prostate-specific antigen; ISUP = International Society of Urological Pathology; pT stage = pathological T stage; pN stage = pathological N stage; PSADT = PSA doubling time; ADT = androgen-deprivation therapy.
