# Supplementary material for: [¹⁸F]PSMA-1007 PET/CT in biochemical recurrence after radical prostatectomy: A single-center experience of detection rate and lesion distribution
Source: PLoS One. 2026 May 14;21(5):e0349397. doi: 10.1371/journal.pone.0349397 (PMC13175320; doi:10.1371/journal.pone.0349397)
Supplement: S4 Table — (DOCX) [file pone.0349397.s004.docx]

**Table S4. Verification Methods of [¹⁸F]PSMA-1007-Avid Lesions (n=184 PET/CT-Positive Patients).**

| **Verification Criterion** | **Number of Patients (n)** | **Percentage of PET-Positive Cohort (%)** | **Number of Discrete Lesions (n)** |
| --- | --- | --- | --- |
| **Histopathological Confirmation** | 59 | 32.1 | 76 |
| **Targeted Therapy + PSA Response** | 48 | 26.1 | 52 |
| **- Targeted Therapy Alone** | 31 | 16.8 | 34 |
| **- Targeted Therapy + Concurrent ADT** | 17 | 9.3 | 18 |
| **Unequivocal Imaging Follow-Up** | 25 | 13.6 | 29 |
| **Total Verified Cases** | 132 | 71.7 | 157 |
| **Unverified Cases** | 52 | 28.3 | — |
| **- Sole Systemic ADT** | 37 | 20.1 | — |
| **- Insufficient Follow-Up (< 3 months)** | 15 | 8.2 | — |

**Abbreviations:** PET/CT = positron emission tomography/computed tomography; PSA = prostate-specific antigen; ADT = androgen-deprivation therapy; SBRT = stereotactic body radiotherapy; SRT = salvage radiotherapy; SLND = salvage lymph node dissection.
